# Near-source high-rate GPS, strong motion and InSAR observations to image the 2015 Lefkada (Greece) Earthquake rupture history

**DOI:** 10.1038/s41598-017-10431-w

**Published:** 2017-09-04

**Authors:** Antonio Avallone, Antonella Cirella, Daniele Cheloni, Cristiano Tolomei, Nikos Theodoulidis, Alessio Piatanesi, Pierre Briole, Athanassios Ganas

**Affiliations:** 1 Istituto Nazionale di Geofisica e Vulcanologia, Centro Nazionale Terremoti, Via di Vigna Murata 605, Rome, 00143 Italy; 2 Istituto Nazionale di Geofisica e Vulcanologia, sezione di Roma1, Via di Vigna Murata 605, Rome, 00143 Italy; 3 Institute of Engineering Seismology and Earthquake Engineering (ITSAK-EPPO), P.O. Box 53, FInikas, Thessaloniki, 55102 Greece; 4 Ecole Normale Supérieure, PSL Research University, Laboratoire de Géologie - UMR CNRS 8538, 24 Rue Lhomond, Paris, 75005 France; 5 National Observatory of Athens, Institute of Geodynamics, P.O. Box 20048, Athens, 11810 Greece

## Abstract

The 2015/11/17 Lefkada (Greece) earthquake ruptured a segment of the Cephalonia Transform Fault (CTF) where probably the penultimate major event was in 1948. Using near-source strong motion and high sampling rate GPS data and Sentinel-1A SAR images on two tracks, we performed the inversion for the geometry, slip distribution and rupture history of the causative fault with a three-step self-consistent procedure, in which every step provided input parameters for the next one. Our preferred model results in a ~70° ESE-dipping and ~13° N-striking fault plane, with a strike-slip mechanism (rake ~169°) in agreement with the CTF tectonic regime. This model shows a bilateral propagation spanning ~9 s with the activation of three main slip patches, characterized by rise time and peak slip velocity in the ranges 2.5–3.5 s and 1.4–2.4 m/s, respectively, corresponding to 1.2–1.8 m of slip which is mainly concentrated in the shallower (<10 km) southern half of the causative fault. The inferred slip distribution and the resulting seismic moment (M_0_ = 1.05 × 10^19^ N m) suggest a magnitude of *M*_*w*_ 6.6. Our best solution suggests that the occurrence of large (*M*_*w*_ > 6) earthquakes to the northern and to the southern boundaries of the 2015 causative fault cannot be excluded.

## Introduction

**Figure 1:**
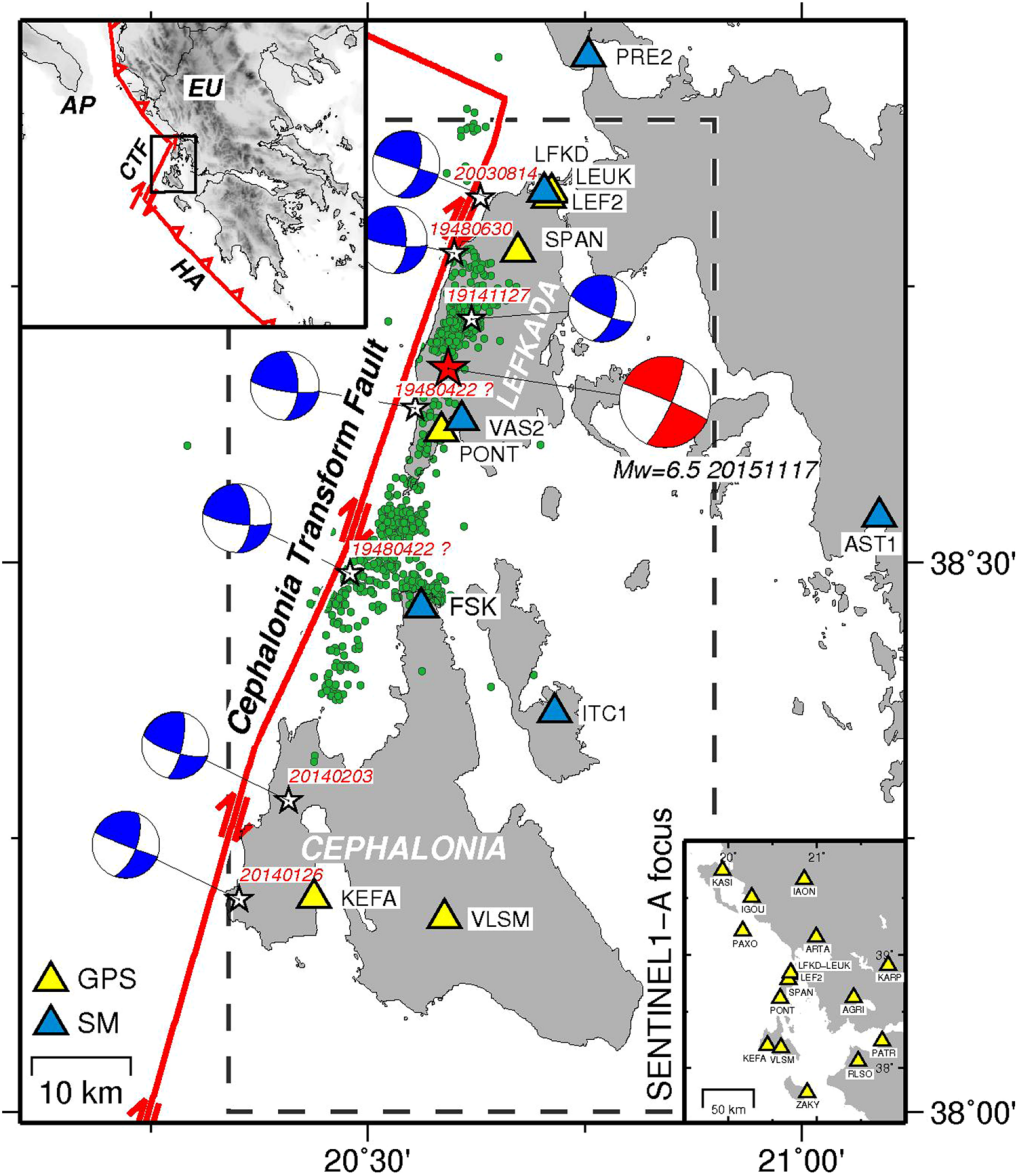
Seismo-tectonic settings of the investigated area. The yellow and blue triangles represent the available GPS and SM stations, respectively, used in this study. The dashed box corresponds to the Sentinel-1A swath subset selected for this study. The green dots are the 956 relocated aftershocks in time interval 2015/11/17-2015/12/30^2^, whereas the red star and the beach ball represent the epicentre^2^ and the USGS focal mechanism^3^, respectively, of the main shock. The blue beach-balls and white stars represent the estimated focal mechanisms and epicentres of the previous largest events (*M*_*w*_ > 6) in the investigated area^13–23^. The major structural discontinuities (red thick lines) are also shown. The inset shows the main tectonic structures: CFT, Cephalonia Transform Fault; HA, Hellenic Arc. The open rectangle indicates the investigated area. Abbreviations: Ap, Apulia; Eu, Eurasia. Map was created using the GMT (Generic Mapping Tools, http://gmt.soest.hawaii.edu/) software package^57^, version 4.5.14.

On 2015 November 17^th^ (07:10:07 UTC), the Lefkada Island (Greece) was struck by an earthquake of magnitude, *M*_*w*_, estimated between 6.4 and 6.6^1–7^, which occurred at a depth^2^ of 9.6 km (Fig. 1). This event produced structural damage and site effects mainly concentrated along the island’s western coast, and acceleration peaks up to 0.36 g in Vassiliki village (strong motion site VAS2)^8^ (Fig. 1). The focal mechanism provided by the USGS^3^ suggests that this event occurred beneath the Lefkada Island, on a near-vertical right-lateral strike-slip fault located close to its western coast. The main shock relocation as well as the relocated aftershocks in the time period 2015/11/17-2015/12/30^2^, are in agreement with the tectonic regime of the area, that is dominated by one of the major active structural discontinuities of the eastern Mediterranean, the Cephalonia Transform Fault (CTF)^9–11^. This fault zone accommodates the relative deformation between the Apulia-Eurasia collision zone on the north and the Hellenic arc subduction zone on the south, with a relative motion of ~20 mm/yr^12, 13^.

In the last century, the CTF zone experienced several moderate magnitude (*M* > 6) earthquakes which occurred both to the NE and the SW of the 2015 epicentre, with focal mechanisms and magnitudes roughly comparable to the one of the 2015 main shock^14^ (Fig. 1). Among the largest historical earthquakes, the 1914/11/27 (*M*_*w*_ 6.3) and the 1948/06/30 (*M*_*w*_ 6.4) events have been located along the western coast of the Lefkada island^14–16^. A higher degree of uncertainty exists for the location of the 1948/04/22 (*M*_*w*_ 6.4) event, that some studies^14, 17–19^ locate close to the 2015 epicentre, whereas others locate it to the SW of the 2015 earthquake^15, 20–22^. More recently, large earthquakes occurred on 2003/08/14 (*M*_*w*_ 6.2)^23–25^ to the NE of the 2015 event, as well as the 2014/01/26 (*M*_*w*_ 6.1) and 2014/02/03 (*M*_*w*_ 6.0) which occurred beneath western Cephalonia^26–28^.

During the 2015 main shock, ground motion signals at broadband seismometers located within a distance of ~80 km from the epicentre (11) were clipped^29^. Due to its location, this event was observed by 12 stations in the near-source (within ~60 km), mostly located along with a profile roughly parallel to the CTF strike (Fig. 1)^9–11^. These stations consist of six Strong Motion (SM) belonging to the Institute of Engineering Seismology and Earthquake Engineering (ITSAK) accelerometric network^8^ and of six continuous GPS (cGPS) stations belonging to different networks developed for both scientific (NOANET^30^) or commercial (METRICANET, SMART-NET Greece and TREE Company SA Greece) purposes. We also considered 10 more distant (>70 km) HRGPS stations (Fig. 1) to better constrain the far field (zero-displacement) area.

The epicentral area was also observed by two Synthetic Aperture Radar (SAR) images’ pairs acquired by the Sentinel1-A satellite in TOPS (Terrain Observation by Progressive Scans) mode for the ascending pass on 2015/11/05 and 2015/11/17 and for the descending orbit on 2015/11/11 and 2015/11/23, respectively. Starting from the original swath area, we focused on an images’ subset of the image (~40 km in W-E × 85 km in N-S) concentrating on the Lefkada Island and surrounding region (Fig. 1).

Definitively, with respect to the previous studies^1–7^, the HRGPS and SM data and InSAR images pairs along both the ascending and descending Lines-Of-Sight (LOS) used in this work represent the most comprehensive data set for the 2015 Lefkada earthquake, allowing us to retrieve a robust image of both the fault geometry and the kinematic rupture model.

Our combined analysis of the 2015 Nov. 17^th^ Lefkada earthquake shows that this event activated a segment of the CTF zone that remained unbroken since at least 1948. Moreover, our best fitting fault modelling suggests that the major CTF discontinuity is slightly closer to the western coast of the Lefkada island, than proposed in previous studies^9–11^. The comparison between the distribution of the largest (M > 6) events along the CTF zone in the last century and our fault modelling of the 2015 main shock suggests that the occurrence of moderate (M6-6.6) earthquakes to the northern and to the southern boundaries of the 2015 causative fault cannot be ruled out.

## Results

**Figure 2:**
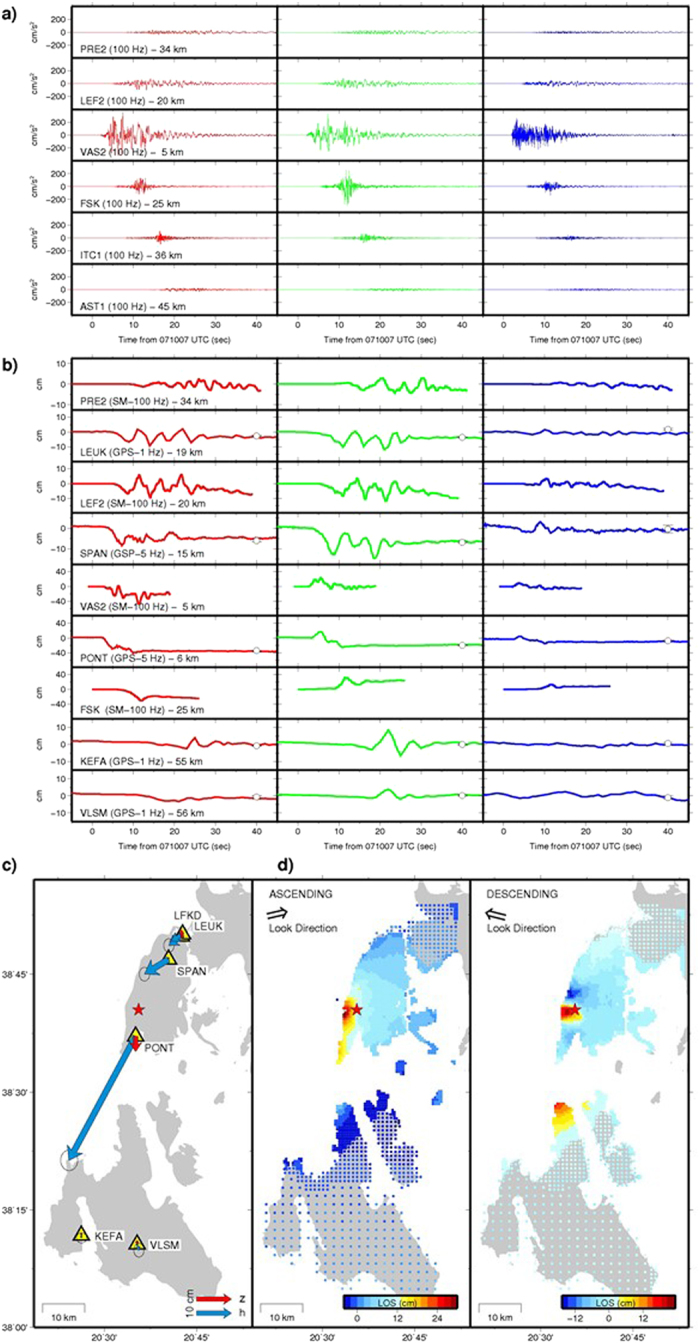
Time histories and static displacements. Time histories of the observed near field accelerations (**a**) and of the high sample rate displacements (**b**) at the available SM and HRGPS sites respectively. The red, green and blue trends represent the North, East and Vertical components, respectively. In the HRGPS time series, the black dashed lines represent the time of the main shock, while the white dots and the vertical bars represent the mean position and their computed 2-σ uncertainties as described in the Supporting Information and in *Ganas et al*.^2^. The labels in (**a**) and (**b**) indicate the name of the site, the sampling frequency and the distance from the epicentre. (**c**) Horizontal (blue vectors) and vertical (red vectors) static offsets of SM and HRGPS sites located in the studied area. The red star indicates the main shock. (**d**) Sub-sampled ascending (left) and descending (right) Sentinel-1A interferograms. The colour palette represents the amount of displacement in the Lines-Of-Sight (LOS). The plot was created using the GMT (Generic Mapping Tools, http://gmt.soest.hawaii.edu/) software package^57^, version 4.5.14.

Among the available HRGPS stations, the two closest sites to the epicentre (PONT and SPAN) recorded the main shock with a 5-Hz sampling frequency, whereas all the others were acquiring at 1-Hz (Fig. 2b), except LFKD station that acquired with a 30-s sampling rate. On the other hand, all the six available strong motion stations in the near field recorded the main shock with a 100-Hz sampling frequency (Fig. 2a). Significant co-seismic dynamic displacements were observed at the near-field HRGPS and SM stations (Fig. 2b). On the other hand, no co-seismic displacements were observed at the more distant (>~70 km) HRGPS stations, which allow us to mainly constrain the far field (zero static displacement) area. The static displacements were retrieved by comparing the mean position before the earthquake to the mean position calculated a few minutes after the event (i.e., the “co-seismic displacements”, Supplementary Methods and Table S1). At station PONT, the ground displacement started 2.5 s after the earthquake origin time (t0), experiencing a three-steps movement, spanning ~8 s before reaching its final static offset. Two major displacement peaks are visible: the first reaching up to ~32 cm in the SE direction and an uplift of ~7.5 cm at t0 + 4 s, and the second reaching up to ~49 cm in the SW direction and a subsidence of ~11 cm at t0 + 10 s. The final static deformation at PONT amounted up to ~40.3 ± 0.8 cm in the S-SW direction with a subsidence of ~4.3 ± 1.2 cm (Fig. 2c). Also at the closest strong motion station VAS2, located ~2 km away from PONT site, the ground motion started at ~t0 + 2.5 s. This site experienced also the highest peak ground accelerations with values up to 0.36 g, 0.32 g and 0.26 g on the NS, EW and vertical components, respectively, as previously proposed^8^.

To the south of the nucleation, at FSK SM station the deformation starts at t0 + 6 s, reaching peaks of displacement up to 29 cm, 26 cm and 12 cm towards the south, east and up directions, respectively, and peak ground accelerations of 0.26 g on the EW component. KEFA and VLSM HRGPS sites (~50 km to the south of the epicentre) appear to be close to the boundary between the near and the far field deformation, as testified by comparable static offsets on either the horizontal and vertical components (Fig. 2c), with values slightly larger than their uncertainties (Table S1). Despite the fact that the start of the ground deformation seems to be synchronous (t0 + 15 s) at both the sites, station KEFA appears to be in a more directive position, experiencing larger dynamic displacements (with peak up to ~16.5 cm in NW-SE direction), than those observed at VLSM site (~7 cm). To the north of the nucleation, SPAN and LEUK stations (~15 to 19 km from the epicentre) observed lower peaks of displacement than PONT site (~16 cm and ~15 cm, respectively, in a NW-SE direction), following directions roughly normal to the CTF zone^9–11^ and to the fault strike of the 2015 event causative source, as proposed by previous studies^1, 2, 4–7^. The static and dynamic deformation shown by LEUK station agree with the almost co-located cGPS site LFKD (Fig. 2c) and with the strong motion LEF2 and PRE2 sites (Fig. 2b), respectively. The static deformation still reaches appreciable values (~4.1 ± 0.3 cm, Table S1) in the W-SW direction (Fig. 2c), whereas the comparison of dynamic deformation between LEUK, LEF2 and PRE2 stations shows 5-s-period nearly harmonic oscillations between t0 + 10 s and t0 + 27 s, with peak-to-peak amplitudes of ~19 cm in the SW-NE direction. This feature suggests a potential characteristic behaviour of the geological Holocene soft outcrops in this part of the island, as testified by liquefactions during the 2003 earthquake main shock^19, 25, 31^ and aftershocks^32^, and from ambient noise analysis^33^.

Finally, the two different SAR interferograms (InSAR) spanning the main shock on both the ascending and descending LOS, even down-sampled to thousands of pixels, allowed an impressive spatial coverage of both the near and far field of the investigated area. The accuracy of these measurements, derived during the data analysis for each pixel, is directly proportional to the coherence value (see Supporting Information) and amounts to values of 0.6 cm for the ascending (Fig. S1a) and 1 cm for the descending (Fig. S1b) interferograms. As expected by the different acquisition geometries of the two images’ pairs, the two interferograms show differences in the retrieved displacement fields (Fig. 2d). The ascending LOS map clearly shows the gradual increase of deformation (positive displacements, i.e. LOS shortening) moving from north and east of Lefkada island and north of Cephalonia island towards the epicentral area, depicting an elongated lobe of major deformation along the south-western coast of the Lefkada island. The maximum displacement is observed to the west of the epicentre and amounts up to ~28.5 cm (LOS shortening). Appreciable displacements (~6 cm) are still observed in the northern part of the Cephalonia island (to the south) and in the Lefkada town (to the north). The descending interferogram shows an apparently different deformation pattern (Fig. 2d). In particular, two close lobes of deformation with reversed sign are observed within an area of ~25 km^2^ (in a SW-NE direction) in the surrounding of the epicentre: one to the north and one to the west, with values of deformation amounting up ~−17 cm (LOS lengthening) and ~25 cm (LOS shortening), respectively. In the descending interferogram, another important lobe of displacement is observed also on the northern coast of the Cephalonia Island, reaching maximum values (~15 cm, LOS shortening) larger than those observed in the ascending track. In the case of Lefkada earthquake, the observed differences in the LOS maps reveal a combination of mainly horizontal movements consistently with the strike-slip mechanism of the CTF zone. However, because of the temporal sampling of the SAR descending images pairs, our nominally co-seismic observation in the descending interferogram potentially will contain some early post-seismic deformation. Nevertheless, we used both the images in the modelling to better constrain fault parameters and co-seismic slip distribution.

In order to image the rupture history of the 2015 Lefkada main shock we used the following three-step self-consistent procedure: firstly, we inverted the static deformation to determine the fault geometry, and then, the best-fit uniform-slip fault parameters are used as *a-priori* for the estimation of the slip distribution. Finally, the resulting finite fault plane was used to determine the rupture process by jointly inverting for static deformation and waveforms. Thus, in the first two steps, we used only data from the two down-sampled interferograms and the GPS co-seismic static offsets. The best-fitting uniform slip model (Fig. S4) is described by a 13°N-striking and 70°ESE-dipping strike-slip (rake about 170°) 27 km × 6 km fault plane, in good agreement with hypocentral location, aftershocks’ distribution, focal solutions and previous uniform slip inversion of comparable geodetic data sets^2^ (Table S3). The derived uniform slip model is characterized by a maximum slip of ~1.3 m, resulting in a seismic moment M_0_ = 0.64 × 10^19^ N m and a moment magnitude of (*M*_*W*_) 6.5, consistent with the Ganas *et al*.^2^ uniform slip model.

In a second step, to test variable slip on the fault plane, we extended the uniform slip fault in order to capture almost the entire area affected by the aftershocks, and discretized the fault into small patches (see Supporting Information). The best-fitting slip distribution on the extended fault model (with a 45 km × 20 km fault plane) is in agreement with the aftershocks distribution (Fig. S5). As expected, the fit significantly improved passing from the uniform slip to a variable slip model: from 2.09 to 1.19 cm for the GPS static offsets, and from 1.38 to 1.33 and from 2.75 to 1.41 cm for LOS displacements on ascending and descending data, respectively. The variable slip model shows two well-separated major asperities with peak of slip of about 2 m, mostly confined within the first 6 km, i.e. roughly contained in the uniform slip fault. In addition, our model shows a third smaller and deeper patch (between 10 and 16 km) to the NE, amounting up to 0.3 m of slip. The total slip distribution and the resulting seismic moment (0.82 × 10^19^ N m) agree with a *M*_*w*_ 6.6 earthquake, in agreement with Saltogianni *et al*.^6^.

**Figure 3:**
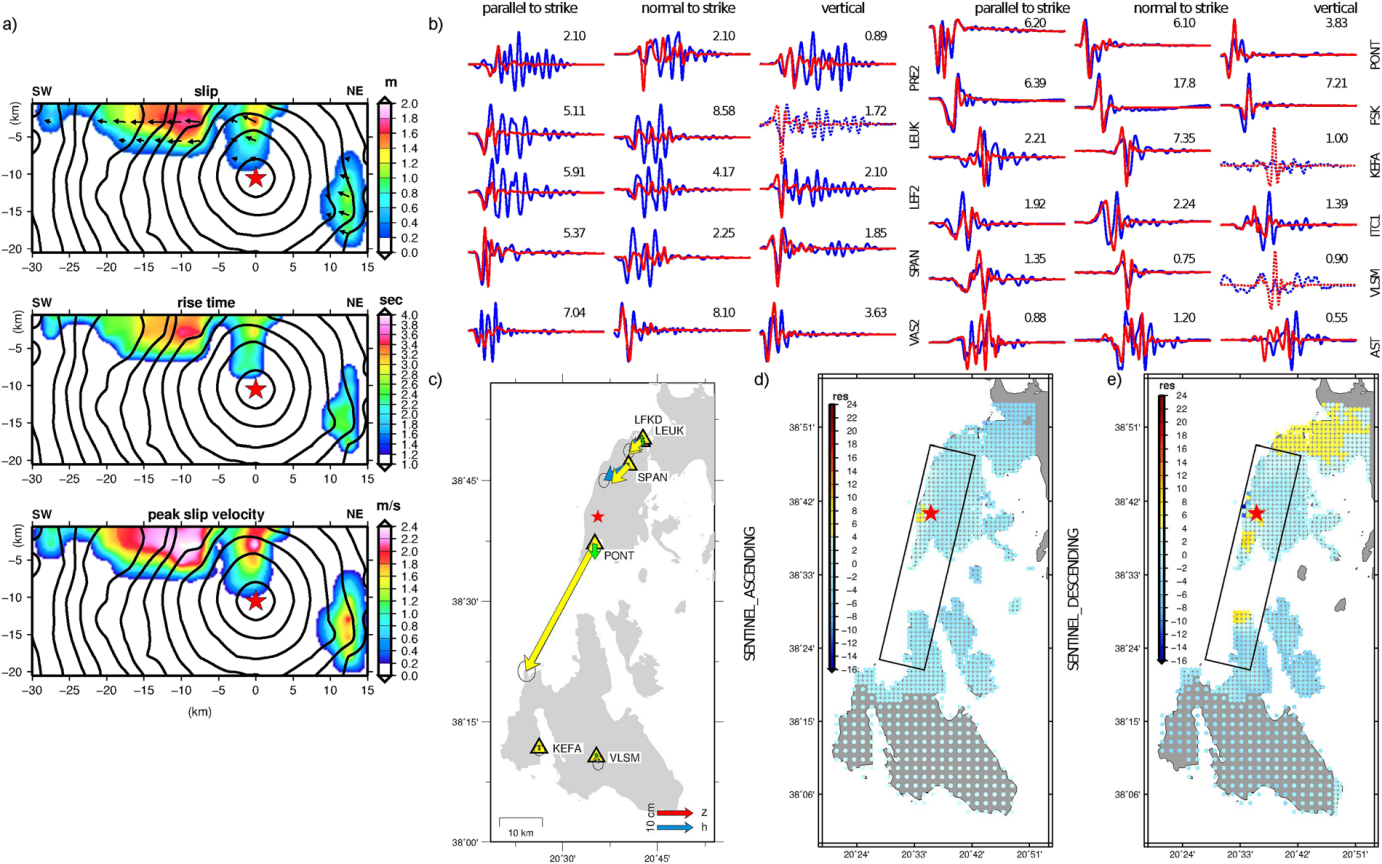
Kinematic rupture model and fit to the data. (**a**) Inverted rupture model of the 2015 Lefkada earthquake. Top, centre and bottom panels display slip, rise time and peak slip velocity, respectively. Black contours yield the rupture time every 1 s; rake angle is shown by black arrows in top panel; (b) comparison of recorded Strong Motion and HRGPS (in terms of ground velocities, blue lines) with synthetic waveforms (red lines). Dashed lines show predicted time series not included during the inversion. Peak amplitudes (cm*s^−1^) of the observed waveforms are given by numbers; (**c**) measured and synthetic horizontal (blue and yellow arrows, respectively) and vertical (red and green arrows, respectively) co-seismic static displacements; (**d**) residuals between the observed and synthetic InSAR displacements, observed from ascending track Sentinel1-A; (**e**) residuals between the observed and synthetic InSAR displacements, observed from descending track Sentinel1-A. The plot was created using the GMT (Generic Mapping Tools, http://gmt.soest.hawaii.edu/) software package^57^, version 4.5.14.

**Figure 4:**
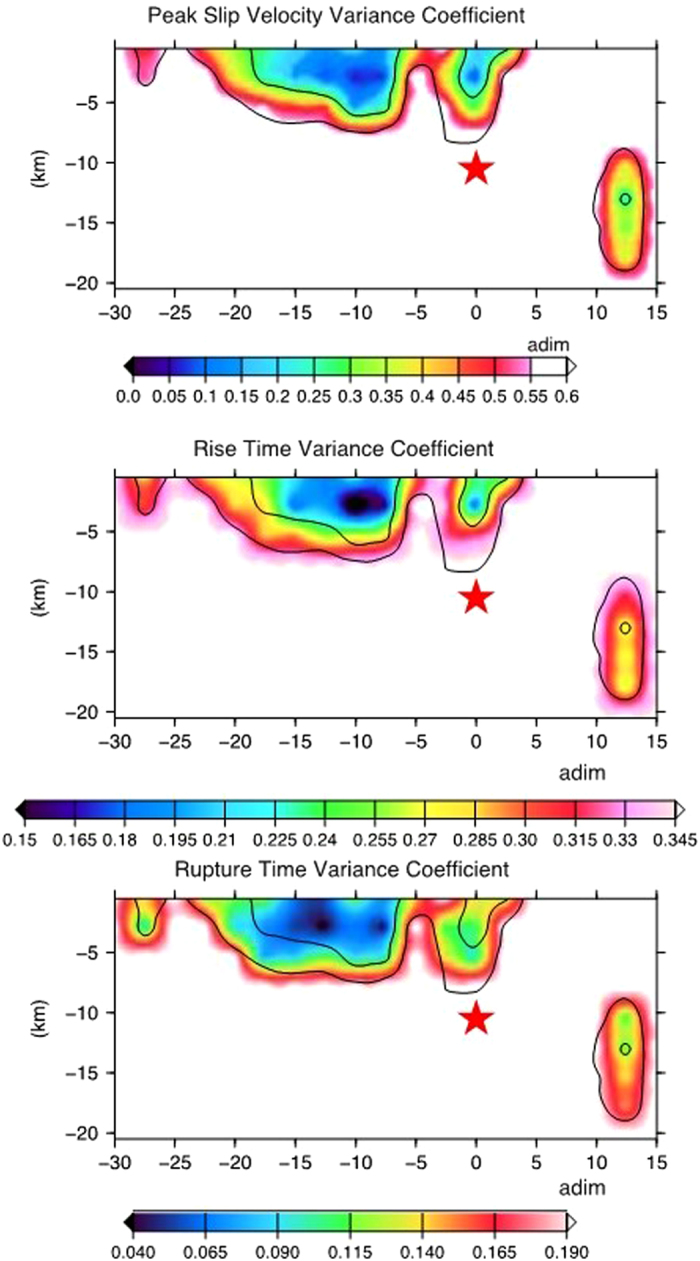
Coefficient of variation (CV). Variance coefficient distribution of peak slip velocity, rise time and rupture time (upper, middle, and bottom panel, respectively) of the 2015 Lefkada rupture model (Fig. 3a) computed through ensemble inference. Black contour lines show the fault region with a peak slip velocity greater than 0.9 and 1.8 m/s. The plot was created using the GMT (Generic Mapping Tools, http://gmt.soest.hawaii.edu/) software package^57^, version 4.5.14.

In the third step, we used both the GPS and InSAR static displacements (Fig. 2c,d), and all the available SM and the near-field HRGPS waveforms (Fig. 2a,b), in our rupture history modelling attempts. Because no significant co-seismic dynamic deformation was observed at the regional (>70 km) HRGPS sites, and after having verified that their contribution to the inverted source model is negligible, we decided to proceed in the modelling excluding them. Despite the limited azimuthal stations’ coverage (~180°), due to the epicentre location (Fig. 1), the InSAR contribution allowed to have a satisfactory data set distribution to image model parameters. The retrieved rupture model (Fig. 3a) was built up by averaging nearly 295000 explored models, corresponding to those models having a cost function exceeding by 2.5% the minimum value of the cost function reached during the inversion. Three principal slip patches characterize the imaged source model: a small patch, located 14 km depth and 12 km along strike to the NE direction from the nucleation point, and two larger and shallower patches located above and 10 km to the SW, respectively, from the nucleation point. These results are in agreement with the results of the inversion of the geodetic data alone. The main shallower asperities are characterized by a rise time of 2.5–3.8 s and by a peak slip velocity of 1.6–2.4 m s^−1^, corresponding to 1.2–1.8 m of slip. The inferred slip distribution and the resulting seismic moment (M_0_ = 1.05 × 10^19^ N m) agree with a moment magnitude *M*_*w*_ 6.6, according with only one of the previous solutions^6^ and with our previous step. The rake angle (Fig. 3a) is consistent with a basically pure right-lateral strike slip faulting mechanism, in agreement with the kinematic slip model proposed by Melgar *et al*.^5^. The rupture velocity (*v*_*r*_) does not change strongly on the fault plane; in fact, we find that the propagation is slightly faster along the up-dip direction (*v*_*r*_ = 3.23 km s^−1^, about 80% of the mean shear wave velocity, Vs, value) than along the strike direction (*v*_*r*_ = 2.56 km s^−1^, about 68% of the mean Vs value). The total rupture duration is ~9 s. The mean model reveals the most robust features of the rupture process. Indeed, the obtained averaged model parameter and the associated standard deviation, computed by weighting all models of the ensemble by the inverse of their cost function values, represent the ensemble properties and are the actual solution of our nonlinear inverse problem. The coefficient of variation (CV, defined as the ratio of the standard deviation to the mean) is useful to measure the dispersion of the model parameters distribution around their respective average values displayed in the average kinematic model. The obtained distributions of the coefficient of variation for rupture times, rise time and peak slip velocity (Fig. 4) reveal a small dispersion of model parameters in the fault portions that slipped during the 2015 Lefkada earthquake. This analysis is useful to identify the rupture’ features well constrained by the data. The smallest and deepest patch to the NE direction, observed also in the second step by using only static deformation (Fig. S5), appears to be not an artefact of the modelling. In fact, the forward modelling from our final solution revealed that this patch is needed to fit the signal observed by the stations located to the north of the nucleation point on all the components (PRE2 site) or only on their vertical one (SPAN, LEUK, LEF2 stations) (Fig. S6). Our source model (Fig. 3b) does not fit the 5-s-period nearly harmonic signal observed at these northern sites in the velocity time histories a few seconds after the first arrivals. However, the fit of the first arrivals at these sites as well as the fit of the overall ground velocity time histories at all the other sites in the near field shows a very good agreement in both phase and amplitude (Fig. 3b). In addition, the horizontal and vertical synthetic static GPS offsets (Fig. 3c) match generally well with the observed ones in both amplitude and direction. Finally, the residuals’ images between the InSAR LOS observed and predicted displacements (Fig. 3d,e) at the considered pixels’ subsets (either for descending or ascending Sentinel-1A tracks), show a general satisfactory agreement, with residuals localised only in the nearby town of Lefkada and in the epicentral area. The generally good fit to both static deformation and waveforms would suggest that the later harmonic signal observed at the northern sites is not related to the source properties but most probably to the occurrence of earthquake-related site effects in the vicinity of Lefkada town as it was observed during the 2003 earthquake sequence^19, 31, 32^.

## Discussion and Conclusive Remarks

**Figure 5:**
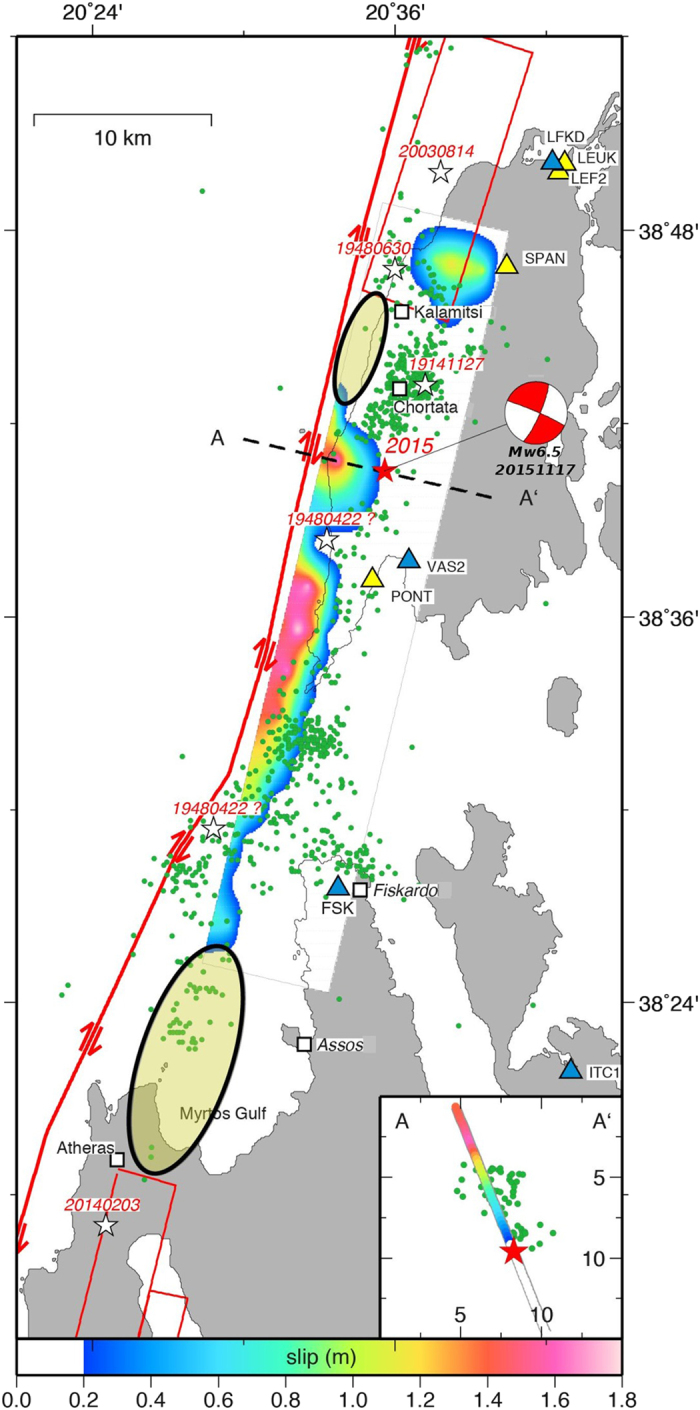
Seismo-tectonic map with inverted rupture model. Inverted rupture model (Fig. 3a) projected on the Earth surface. Colours on the fault plane indicate the slip distribution. The red star and the beach ball show the location of the main shock^2^ and the USGS focal mechanism^3^. The green dots represent the 956 relocated aftershocks in the time period 2015/11/17-2015/12/30^2^. The red fault boxes shown to the NE and to the SW of the 2015 Lefkada causative fault (this study) represent the locations proposed for the faults related to the previous Lefkada August 14, 2003^24^ and the Cephalonia January 26^th^ and February 3, 2014^25, 26^ earthquake sequences, respectively. The inset shows the estimated slip distribution as function of depth (symbols as in the main figure). The black yellow-shaded ellipses correspond to slip deficit areas as suggested in this study. Map was created using the GMT (Generic Mapping Tools, http://gmt.soest.hawaii.edu/) software package^57^, version 4.5.14.

The estimation of the slip distribution and rupture history of the 2015 Lefkada earthquake was performed following a self-consistent three-step approach: using only static deformation, the first two steps dealt with retrieving the best-fit parameters of the finite-fault geometry and the variable slip distribution on the fault plane. In the final step, the extended fault model was used in a joint non-linear inversion of both static deformation and near-source HRGPS and SM waveforms. Our preferred final model predicts a bilateral rupture propagation with the activation of three main patches of slip (Figs 3a and 5), a smaller and deeper (~14 km) patch to the NE of the nucleation point, and two larger and shallower (within 8 km) patches related to the southern half part of the causative fault, where most of the slip (up to 1.8 m) was released. The two main shallow asperities characterizing our rupture model agree with the up-dip rupture episodes and with the SW rupture directivity, as retrieved and discussed by previous studies^1, 5, 7^. The third smallest patch, located to the NE of the nucleation, implies instead a bilateral rupture propagation, in agreement with the model proposed by Chousianitis *et al*.^4^. This latter study suggests a 18-km-long and 8-km-downdip-wide fault portion activating the two main patches, in agreement with the portion of our model where most of the slip occurs (17–18-km-long and 8-km-wide, Fig. 5). On the other hand, a longer activated fault portion is suggested in the Sokos *et al*.^1^ (~25 km), and in Melgar *et al*.^5^ and Bie *et al*.^7^ studies (~30 km). The main difference between our results and all the previous studies is basically related to the joint use of information on both dynamic and static deformation: the HRGPS and SM waveforms allows us to mainly constrain the time history and the deeper NE patch; the HRGPS-derived static offsets as well as the inclusion of both the ascending and the descending tracks provide a crucial contribution to better constrain the finite fault rupture model and to supply a good azimuthal coverage in the near source region. Furthermore, those last observations (Fig. 2c,d) allowed us to robustly constrain the finite-fault geometry (Fig. S3) to significantly lower dip and strike angle values with respect to previous studies^1, 4^, in agreement with recent studies based on the use of dense InSAR data^2, 5, 7^. In contrast to Chousianitis *et al*.^4^ and Bie *et al*.^7^, our model features a predominantly right-lateral strike-slip mechanism without significant rake variations on the fault plane. The maximum slip (1.8 m) in our preferred model is comparable with some of the previous studies proposing 1.5 m^1^ or 1.6 m^5, 7^, and it occurred on the south-westernmost patch in agreement with Chousianitis *et al*.^4^, although it is significantly lower than the maximum slip modelled by this study (2.35 m).

The distribution of the coefficient of variation for the kinematic parameters retrieved for the 2015 Lefkada earthquake (Fig. 4), reveals small dispersion in the fault portions that mostly slipped during the event. This suggests that the average model contains stable features of the rupture history. This approach represents a key contribution to the nowadays studies on this earthquake and it suggests that the source model proposed in this work contains stable features of the 2015 Lefkada rupture history, allowing us to characterize its relevant properties, and to assess the corresponding uncertainties. In particular, we emphasize that the CV associated to the smallest and deepest patch located to the NE part of the fault plane, indicates a small dispersion, comparable to the CV associated to the shallower slip patch suggesting that the resolution available with the used data set allows us to robustly constrain this rupture feature, by corroborating the forward modeling results, described in the previous section.

The comparison between the observed and modelled GPS-derived static deformation field is quite satisfactory within the accuracy of the measurements. Moreover, the fit to InSAR data show residuals generally confined to within ±3 cm, except on the north-eastern and south-western part of the Lefkada coast. These higher residuals could be mainly due to the presence of early post-seismic deformation in the signal, due to the 2^nd^ image of the descending track acquired six days after the main shock occurrence (on 2015 Nov. 23^rd^). Starting from the available data, we performed a preliminary analysis to investigate any potential evidence of post-seismic deformation at least at the cGPS sites located near the source. The static offsets computed using the position the day before the event and the one retrieved for periods up to 6 days (depending on the available data) are named “co + post-seismic” displacements (Table S2). The projection of the “co-seismic” and of the “co + post-seismic” displacements in the LOS of the ascending and descending interferograms, respectively (Fig. S7a,b), revealed a general agreement between the GPS and InSAR displacements, with correlation coefficients of 0.96 and 0.94 for the ascending and descending track, respectively. In particular, the comparison between the two independent datasets show that the residuals in Lefkada town area (LEUK and LFKD sites) and in the epicentral area (close to station PONT) in the descending track (Fig. S7b) are consistent with the occurrence of an early post-seismic deformation.

Our best fitting finite fault model suggests that the major CTF discontinuity in the Lefkada area (Fig. 4) could be slightly closer to the western coast of the island than proposed in previous studies^9–11^, which has strong implications in terms of seismic hazard in the investigated area.

The 2015 Nov. 17^th^ Lefkada earthquake finite-fault modelling indicates that this event activated a segment of the CTF zone, which has not ruptured in the recent past^1, 4^. However, the locations of the 1914 and of the 1948 events should be taken with caution because of the scarcity of the available data. It is possible that the 1948 April 22^nd^ and the 2015 earthquake ruptures partially overlap in space. Thus, we conclude that the 2015 Lefkada event ruptured a segment of the CTF zone which remained unbroken since at least 1948. The length of this CTF segment and the estimated magnitude are comparable to the previous largest earthquakes occurring in the investigated area in the last century^15^ (Fig. 5), thus confirming previous hypothesis of a segmented CTF zone able to activate 20–40 km-long segments, corresponding to M 6–7 class earthquakes^9, 14, 26^.

The slip distribution carried out by our modelling is in good agreement with the distribution of the previously proposed relocated aftershocks^2^ (Fig. 5) and a clear anti-correlation can be observed. To the north-east, an aftershocks cluster occurs between the deeper and smaller northernmost patch of slip and the larger and shallower one located close to the nucleation point, whereas, to the south-west, the aftershocks are distributed in an apparently broader area between the southernmost patch of slip and the Myrtos Gulf, on the northern coast of the Cephalonia island.

The comparison between our best-fitting finite-fault modelling and the locations of the finite faults suggested for the 2003 Lefkada^25^ and 2014 Cephalonia^26, 27^ seismic sequences, highlights some interesting features in terms of seismic hazard (black yellow-shaded ellipses in Fig. 5). To the north of the 2015 epicentre, with respect to Bie *et al*.^7^, although our finite-fault partially overlaps the 2003 causative fault, our final slip distribution suggests an absence of slip occurred in the ~7–10-km-long and 8–10 km-wide fault portion existing between the patch located above the 2015 nucleation and the southern limit of the 2003 fault. Using the empirical relationship of Wells and Coppersmith^34^, this gap would correspond to a potential earthquake of *M*_*w*_ 6.1–6.3 in the area between the villages of Chortata and Kalamitsi, and this estimation is in agreement with Ilieva *et al*.^25^ (Fig. 5). Furthermore, referring to the location of our NE deeper patch, this gap could be also larger (up to *M*_*w*_ 6.5) if the shallower NE fault portion didn’t rupture during the 2003 event. To the south of the 2015 epicentre, some authors^22–24^ proposed the occurrence of a second significant (in terms of slip produced) sub-event of the 2003 Lefkada earthquake sequence with an estimated *M*_*w*_ ~5.8–6^1, 22, 23^, roughly in the region probably activated by the 1948/04/22 event. On the other hand, this second event is still a matter of debate. In fact, other studies^11, 14, 18, 21^ attribute this cluster to triggered seismicity due to stress increase from the 2003 main shock with respect to typical aftershocks activity. More recently, Briole *et al*.^26^ suggested that the total length of the proposed ruptured segments would not be compatible with the seismic moment released by the 2003 sequence. Our results show that this region experienced a still significant (up to 0.8 m) and shallow (<5 km) slip during the 2015 earthquake. The slip model obtained by Bie *et al*.^7^ shows some significant slip (~1 m) in the southern fault part, corresponding to the Myrtos Gulf region but they also sentence that this patch is not reliable because it is located in a poorly resolved area. Thus, the southern limit of our finite-fault could represent the northern boundary of a 15–20-km-long region, extending southwards until the 2014 Cephalonia causative faults northern boundaries (in the Myrtos Gulf, offshore the villages of Fiskardo, Assos and Atheras), which could correspond to a potential earthquake of *M*_*w*_ 6.4–6.6 using the known regression of rupture length and moment magnitude^34^.

We are aware that, in general, the seismic gaps could not represent a condition for the occurrence of new earthquakes. On the other hand, the fact that similar magnitudes and mechanisms are observed for the earthquakes of the CTF zone^14^ cannot be ignored. For this reason, our results suggest that the occurrence of a Mw 6.1–6.3 earthquake in the Chortata and Kalamitsi area (North of the 2015 epicentre) and of a Mw 6.4–6.6 event to the north or northwest of Assos-Fiskardo region (Northern Cephalonia island) cannot be ruled out, and therefore a significant seismic hazard still remains in the area.

## Methods

### High-rate GPS data analysis

The HRGPS data analysis was performed following the Precise Point Positioning (PPP) strategy^35^ by using the Gipsy-Oasis II software (v6.4) released by Jet Propulsion Laboratory (JPL, http://gipsy-oasis.jpl.nasa.gov) and the JPL final orbits and high-rate (30-s) clocks products. In this study, the PPP method was applied to ionosphere-free carrier phase and pseudorange data and a 2^nd^ order ionospheric correction was estimated by using the IONEX ionosphere model^36^. We applied the VMF1 grids mapping function to estimate every epoch tropospheric wet zenith delay and horizontal gradients as stochastic random-walk parameters to model troposphere refractivity^37^. We also applied the FES2004 tidal model coefficients provided by the Ocean Tide Loading Provider (http://holt.oso.chalmers.se/loading) to model ocean loading^38^. Finally, we used the absolute antenna calibration to improve the model of the GPS antennas, and the epoch-by-epoch ambiguity resolution by using the wide-lane and phase bias (WLPB) method^39^. In practice, the JPL final fiducial orbits and high-rate (30-s) clocks were held fixed to estimate receiver clocks, epoch-by-epoch random walk zenith troposphere delay and random walk receiver positions directly in the IGS08 reference frame^40^. We also applied to the resulting HRGPS time series a sidereal filtering following Choi *et al*.^41^ to minimize the multipath effect that can affect the static offsets estimations. The estimation of the static offsets derived from the HRGPS time series is described in the Supplementary Information.

### Strong motion data analysis

Starting from the unfiltered acceleration time histories, we retrieved the displacement time series for stations located closest to the source (VAS2, FSK, LEF2 and PRE2) in order to add information on the dynamic deformation near the source from another independent dataset. For this purpose, a 20-s window was selected starting from the very first arrival of P-waves and including S-waves, which is the most energetic part of the recording. Double integration was performed by means of *Geodas* ^42^ and *GSAC* ^43^ software, independently.

### InSAR data processing

Two co-seismic Sentinel-1A (operated by the European Space Agency, ESA) interferograms were generated along both the ascending and descending orbit. We considered SAR acquisitions in TOPS mode using the Sarscape© processor^44^. We adopted the 1-arcsecond (30 m) SRTM digital elevation model from the Shuttle mission (http://www2.jpl.nasa.gov/srtm) to co-register the SAR images, to remove the topographic phase contribution and for the final geocoding. A 1 × 6 multi-looking factor was applied in the azimuth and range direction respectively to resample the SAR images, resulting in a final ground resolution of 25 m. The first-order orbital effect was removed by using the precise orbit files delivered by ESA. Moreover, we chose a set of ground control points in highly coherent areas in the far field to estimate and remove any second-order plane, thus retrieving the corrected LOS maps. A filtering algorithm^45^ was applied before adopting the Delaunay Minimum Cost Flow algorithm to unwrap the interferograms^46^. Being aware that phase unwrapping in coherent areas (i.e. islands) separated by incoherent regions (i.e. sea) could be difficult and challenging, an assessment of the interferograms unwrapping is described in the Supporting Information (Fig. S2). A resolution-based algorithm was used to down-sample the interferograms before modelling, sampling the interferograms more densely close to the epicentral area^47, 48^.

### Finite-fault modelling

We performed the modelling of the static deformation using rectangular dislocations in an elastic, homogeneous and isotropic half-space^49^. The source modelling was carried out with a standard two-step inversion procedure^50, 51^: a non-linear optimization of the fault geometry with uniform slip followed by a linear inversion for slip distribution on the fault with best-fit fixed geometry, discretized into small fault patches (see the Supporting Information for the details). Additional terms consisting of a linear ramp for each SAR interferogram are also included in the inversion. The weighting matrix used in the inversion of the static displacements is built with the weighting matrices of every data set (See Supporting Information for the details). Finally, in the last step, according to the adopted inversion approach (see Piatanesi *et al*.^52^ and Cirella *et al*.^53^ for details), the original strong motion acceleration and HRGPS displacements recordings are integrated and differentiated, respectively, to obtain ground velocity time histories. The resulting velocity waveforms are band-pass filtered between 0.02 and 0.45 Hz using a two-pole and two-pass Butterworth filter. We inverted 60 seconds of each waveform, including body and surface waves. According to the results obtained from the only-geodetic inversion, we performed the joint inversion assuming the fault plane parameters derived in the first step (N13°E strike, 70° dip in the SSE direction and the east position, Fig. S4) and in the second step (finite fault dimensions, 45 km × 20 km, and north position, Fig. S5). We performed the inversion assuming a rupture starting point, consistent with the relocated hypocentre proposed by Ganas *et al*.^2^ (lat 38.68 N, long 20.59E, depth 9.6 km). We use a well-known two-stage non-linear inversion technique (Cirella *et al*.^53^, and references therein) able to jointly invert waveforms (SM and HRGPS) and static deformation (GPS and InSAR offsets). The InSAR observations are inverted along their relative LOS. We applied the same global optimization procedure, a heat bath simulated annealing algorithm, to invert all the data sets, but we start in adopting different cost functions for the strong motion, HRGPS, and static displacements. The total cost function was computed through a weighted sum of the cost functions associated to the different datasets. However, after several ‘trial-and-error’ tests, we found reasonable, for this study, to maintain the same weight for all datasets. We invert for four source parameters, which are spatially variable on the fault plane: peak slip velocity, slip direction, rupture time and rise time. The final slip distribution is computed from inverted model parameters (rise time and peak slip velocity) according to the assumed source time function. In this study, the adopted source time function is the regularized Yoffe function^54^ having a constant time to peak slip velocity (T_acc_) equal to 0.225 s and all kinematic parameters are simultaneously inverted at nodal points every 2.5 km equally spaced along strike and dip. During the inversion, the peak slip velocity is allowed to vary between 0 and 2.5 m/s with 0.25 m/s step increment and the rise time between 1 and 4 s with 0.25 step increment; the rake angle ranges between 150° and 190° with 5° step increment and the rupture time distribution is constrained by a rupture velocity ranging between 2 and 4 km/s. The Green’s function on the fault plane are computed using the Discrete Wavenumber – Finite Fault (DWFE) integration technique implemented by Spudich and Xu^55^ taking into account the complete response of a 1-D layered medium, and assuming the velocity structure proposed by Haslinger *et al*.^56^.

## Electronic supplementary material

Supporting Information
