# Supplementary material for: Near-source high-rate GPS, strong motion and InSAR observations to image the 2015 Lefkada (Greece) Earthquake rupture history
Source: Sci Rep. 2017 Sep 4;7:10358. doi: 10.1038/s41598-017-10431-w (PMC5583176; doi:10.1038/s41598-017-10431-w)
Supplement: Supplementary file 1 — Supporting Information [file 41598_2017_10431_MOESM1_ESM.pdf]

Supporting Information for

Near-source high-rate GPS, strong motion and InSAR observations to image the 2015 Lefkada (Greece) Earthquake rupture history

Antonio Avallone[1,*], Antonella Cirella[2], Daniele Cheloni[1], Cristiano Tolomei[1], Nikos Theodoulidis[3], Alessio Piatanesi[2], Pierre Briole[4] and Athanassios Ganas[5]

[1]Istituto Nazionale di Geofisica e Vulcanologia, sezione Centro Nazionale Terremoti, Rome, Italy
[2]Istituto Nazionale di Geofisica e Vulcanologia, sezione di Roma1, Rome, Italy
[3]Institute of Engineering Seismology and Earthquake Engineering, Thessaloniki, Greece
[4]Ecole Normale Supérieure, Paris, France
[5]National Observatory of Athens, Athens, Greece
*Corresponding author: antonio.avallone@ingv.it

1. Supplementary Methods

High-rate GPS time series and "co-seismic" static displacements: The Figure 2b (main text) shows the HRGPS time series for the sites for which significant displacements have been detected (PONT, SPAN, LEUK, KEFA and VLSM). At the regional HRGPS stations (> 70 km from the epicenter) no significant coseismic displacements were observed. Starting from the HRGPS time series, the static displacements ("co-seismic" displacements) were obtained computing the average positions and the relative standard deviation in a 200-s time window before and after the earthquake[1]. We ignored a 400-s time interval to avoid that the static offsets estimation is affected by the coseismic dynamic displacements. The same procedure was applied to the 300-s-sampled LFKD time series. The derived static offsets are shown in Table S1.

Accuracy of the InSAR measurements and unwrapping assessment: The estimated accuracy of InSAR LOS measurements depends on the interferometric coherence values (γ) and the used wavelength (λ) and was computed during the analysis[2] by using the following formulation:

$$\sigma = \sqrt{\frac{1-\gamma^2}{2\gamma^2}}\,\frac{\lambda}{4\pi} \qquad (1)$$

The histogram distributions of the LOS measurements accuracy (Fig. S1) for the ascending and descending track show mode values of 0.3 cm and 0.5 cm, respectively. Hereinafter, we will take into account the 95% of confidence (i.e. 2-σ), thus resulting in final accuracies of 0.6 cm for the ascending track and 1 cm for the descending one.

Phase unwrapping in coherent areas (i.e. islands) separated by incoherent regions (i.e. sea) is known to be difficult and challenging. To assess the unwrapping carried out on the whole selected scene in this context (Fig.1), we performed two others unwrapped interferograms by selecting alternately only one of the two islands. Thus, we produced three independent unwrapped interferograms for both ascending and descending tracks: Unwrapped Interferograms of including both Lefkada and Cephalonia islands (named "*UILC*") (Fig.S2a,e); Unwrapped Interferogram including only Lefkada Island (named "*UIL*") (Fig.S2b,f); Unwrapped Interferogram including only Cephalonia Island (named "*UIC*") (Fig.S2c,g). The comparison between the LOS displacement values obtained along profiles in each unwrapped interferogram (Fig.S2d,h) shows an agreement

between the different unwrapping solutions within the accuracy (dashed lines of Fig.S2d,h) of the InSAR measurements.

Modeling of the static deformation: To infer the geometry of the uniform slip fault we modeled the observed coseismic displacements, using a rectangular dislocation in an elastic, homogeneous and isotropic half-space[3]. As the displacements are related nonlinearly to the nine parameters describing the geometry and the slip of the fault plane, we used a simulated annealing algorithm[4] to determine the model that produces the least squares best fit. To estimate the confidence intervals, we used a Monte Carlo simulation technique[5] that applies the best-fitting optimization to a large number of synthetic data sets (in this study we performed 500 synthetic data sets), each one derived from adding synthetic realizations of data noise to the actual data set. The individual model parameters appear to be well resolved with compact 95% of confidence intervals (Fig. S3) and consistent with Ganas *et al.*[1]. In a second step, to estimate the variable slip distribution on the fault plane, we discretized the best-fit fault derived from uniform-slip modeling into smaller patches of 2 km x 2 km. We then extended the fault from the surface to a down-dip depth of about 20 km and along-strike up to 45 km in order to capture the entire area affected by the LOS displacement pattern (Figs. S4 and S5). Additional terms consisting of a constant offset and a linear ramp for InSAR displacements are also included in the inversion to minimize the effect of any residual long-wavelength orbital signal in the interferograms. The linear inversion was carried out using a bounded-value, weighted least squares algorithm[5], to impose bounds on the estimated slip and on the nuisance parameters (i.e., the ramp gradient and constant offset). The linear inversion included also a smoothing constraint that is imposed using the Laplacian operator with a scalar smoothing factor and a weight matrix that contains the matrices of weights for each data set (GPS and InSAR data, respectively). In particular, the GPS data are weighted by their inverse covariance matrix, while the InSAR data are assumed to be independent and have constant data variances within a data set (assumed variance of each measurements is 1 cm^2). In addition, for this study, we found reasonable to maintain the same relative weight between the two different data set. Indeed, this choice does not substantially degrade the quality of fit for one data set respect to the other data set.

Kinematic parameters range of variability: All the kinematic parameters are inverted at each nodal point. During the inversions, the ranges of variability of these parameters are a-priori assigned and are listed in Table S4.

Stable Features of Rupture Model: We investigated the robustness of the third small slip patch, located to the NE direction on the fault plane, retrieved from the joint inversion (see Figure 3a in the main text). We performed a forward modeling by selecting only this smallest asperity in order to quantify its contribution to the radiated seismic energy. In Figure S6, we compare the observed waveforms to the modeled and the forwarded ones (blue, red and green lines, respectively) as predicted by the third small patch. The Figure S6 shows that the latter asperity is needed in order to fit the velocity time histories associated to the north-eastern sites (SPAN, LEUK, LEF2, PRE2). This suggests that the average model contains stable features of the rupture history, confirming that the smallest asperity inferred northeast of the epicenter, is not an artifact, but a robust feature that implies bilateral rupture propagation.

Estimation of GPS "co+post-seismic" displacements and comparison with SAR: We determined the static displacements for time intervals as comparable as possible to the time spanning of the descending interferograms (t0+6days, "co+post-seismic" displacements). Thus, we carried out GPS static-mode daily position time series by using the same strategy of the HRGPS data analysis (see Methods of the main text). The "co+post-seismic" static displacements were determined comparing the daily position time series estimated on Nov. 23rd (for PONT, LFKD and VLSM) or on the last available date (for SPAN, LEUK and KEFA) to the one estimated the day before the

earthquake (Nov. 16th) (Table S2). The accuracy considered in Table S2 correspond to the higher value (larger uncertainty), with 95% of confidence, between the uncertainty of the pre-seismic solution and the uncertainty of the post-seismic solution. To compare the InSAR measurements with the GPS-derived static deformation, we averaged the LOS displacements values within a radius of 100 m from the position of the GPS site. Then, we projected the "co-seismic" and the "co+post-seismic" offsets into the LOS of the ascending (Fig. S7a) and descending (Fig. S7b) tracks, respectively.

2. Supplementary Tables

Supplementary Table S1: Static deformation observed at HRGPS and SM sites closer to the source. Static deformation observed at HRGPS sites closer to the source: Mean co-seismic offsets (A), for each component ($_{n,\,e,\,v}$), and relative 1-sigma uncertainties (σ) calculated before (^{b}) and after (^{a}) the main shock (see main text). Number of samples (N) used in each average and acquisition sampling rates (S), as well as site coordinates (Lat, Long) are also shown.

SITE	Lat (°)	Long (°)	A_n (cm)	σ_n^b (cm)	σ_n^a (cm)	A_e (cm)	σ_e^b (cm)	σ_e^a (cm)	A_v (cm)	σ_v^b (cm)	σ_v^a (cm)	N^b	N^a	S (Hz)
KEFA	38,20	20,44	-0.7	0.4	0.3	0.0	0.2	0.3	0.7	0.5	0.8	201	201	1.0
LEUK	38,83	20,71	-2.6	0.4	0.4	-3.6	0.2	0.3	-1.5	0.6	0.8	201	201	1.0
LFKD	38,83	20,71	-2.0	0.1	0.0	-3.3	0.1	0.0	-1.4	0.4	0.0	10	10	0.003
PONT	38,62	20,59	-35.5	0.6	0.5	-19.1	0.4	0.5	-4.3	1.2	1.1	1005	1005	0.2
RLSO	38,06	21,46	0.2	0.4	0.3	-0.2	0.3	0.4	2.2	0.6	0.7	201	201	1.0
SPAN	38,78	20,67	-4.2	0.4	0.4	-6.9	0.2	0.3	0.6	0.7	0.9	1005	1005	0.2
VLSM	38,18	20,59	-1.7	0.4	0.4	0.5	0.3	0.4	1.2	0.7	1.6	201	201	1.0

Supplementary Table S2: Static displacements for the post-seismic period. Static offsets observed at GPS sites closer to the source in the time interval November 16th and November 23rd. For the sites SPAN, LEUK and KEFA GPS data, text in the suffix indicates the number of days after the main shock used for the estimations of co+post-seismic displacements.

SITE	Lat (°)	Long (°)	A_n (cm)	σ_n (cm)	A_e (cm)	σ_e (cm)	A_v (cm)	σ_v (cm)
KEFA^{+2d}	38,20	20,44	-0.9	0.3	-0.8	0.16	0.13	0.50
LEUK^{+2d}	38,83	20,71	-2.9	-3.3	-0.6	0.17	0.13	0.52
LFKD^{+6d}	38,83	20,71	-2.8	-2.6	0.2	0.18	0.15	0.60
PONT^{+6d}	38,62	20,59	-38.1	-20.5	-6.6	0.20	0.18	0.80
SPAN^{+3d}	38,78	20,67	-5.8	-7.2	-1.5	0.19	0.16	0.66
VLSM^{+6d}	38,18	20,59	-1.9	0.8	-0.5	0.19	0.17	0.70

Supplementary Table S3: Best-fit fault parameters determined from uniform slip inversion of geodetic data.

	**Lon* (deg)**	**Lat* (deg)**	**Depth (km)**	**L (km)**	**W (km)**	**Dip (deg)**	**Strike (deg)**	**Rake (deg)**	**Slip (m)**	**Mo (10^{19} Nm)**	**Mw**
This study	$20.523^{-0.9}_{+0.5}$	$38.594^{-0.5}_{+0.6}$	$0.00^{-0.0}_{+0.5}$	$27.8^{-1.1}_{+1.0}$	$5.9^{-1.3}_{+1.5}$	$70.1^{-14.6}_{+11.5}$	$13.1^{-3.5}_{+3.6}$	$169.0^{-6.3}_{+2.2}$	$1.4^{-0.3}_{+0.4}$	$0.71^{-0.3}_{+0.4}$	6.5
Ganas *et al.*[1]	20.520	38.600	0.15	23.00	10.00	71.0	18.0	180.0	1.20	0.93	6.5

* Geographic coordinates of the center of the upper edge of the fault.

Supplementary Table S4: Kinematic parameters' range of variability.

Kinematic parameters	Min Value	Max Value	Step Increment
Peak Slip Velocity [m s^{-1}]	0	2.5	0.25
Rise Time [s]	1	4	0.25
Rake Angle [deg]	150	190	5
Rupture Velocity [km s^{-1}]	2	4	0.25

3. Supplementary Figures

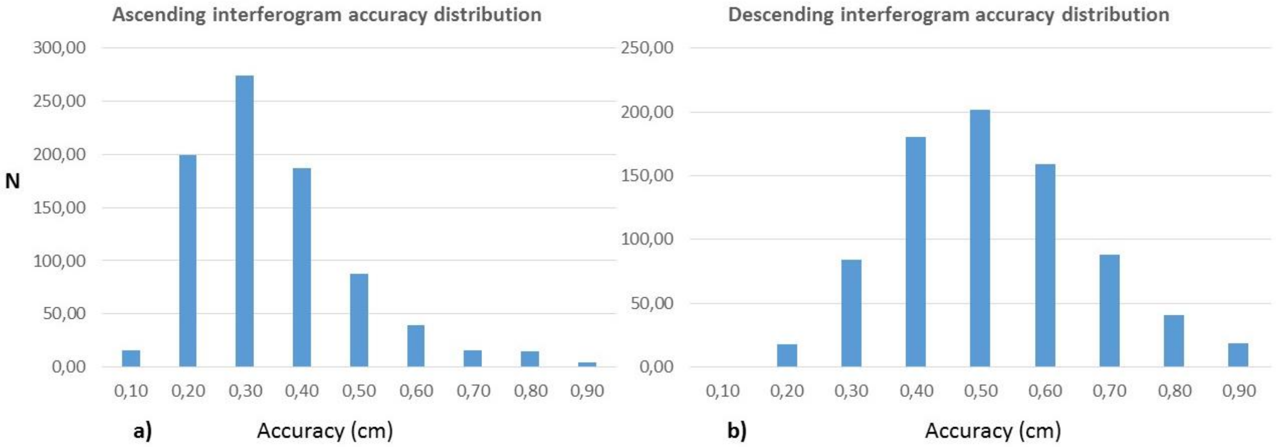

Supplementary Figure S1: SAR accuracy distribution. Histogram distribution of the LOS displacements accuracy at each pixel, computed as described in the Supplementary Material, in both the ascending (a) and descending (b) interferograms. The plots were created using the Microsoft Excel 2010 package.

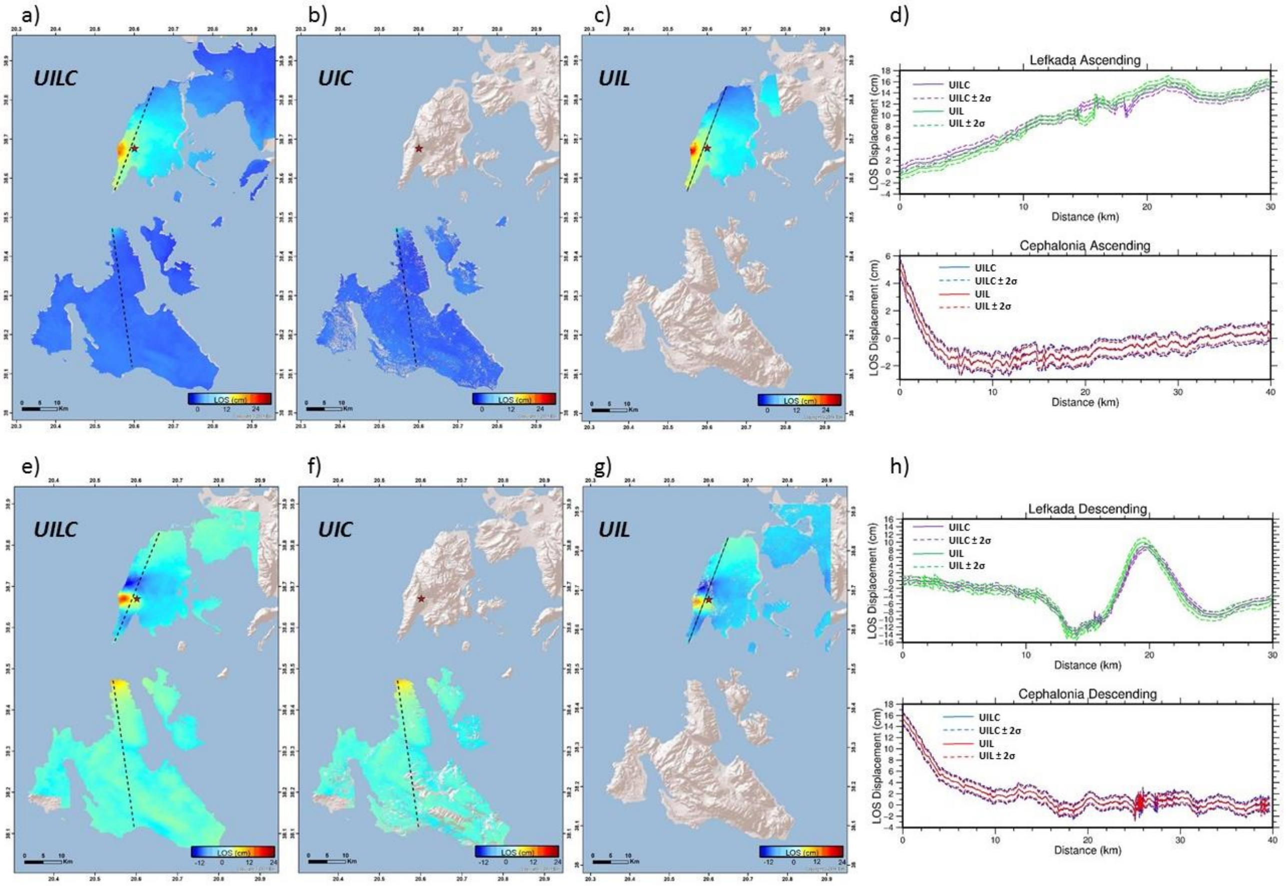

Supplementary Figure S2: Unwrapping assessment. For both the ascending (Fig. S2a-c) and descending (Fig. S2e-g) tracks, we show the Unwrapped Interferograms including both Lefkada and Cephalonia Islands ("*UILC*") (Fig. S2a,e), the Unwrapped Interferograms including only the Cephalonia Island ("*UIC*", Fig. S2b,f), the Unwrapped Interferograms including only the Lefkada Island ("*UIL"*, Fig. S2c,g). The trends plotted for the ascending (Fig.S2d) and the descending (Fig.S2h) tracks for Lefkada (Fig.S2d,h-top) and Cephalonia (Fig.S2d,h-bottom) islands represent the LOS displacements values (continuous colored lines) and their 95% of confidence (dashed colored lines) along the profiles plotted on each island and each interferogram (black dashed lines in the maps). Maps (Fig.S2a-c,e-g) were created using the ArcGIS Desktop (ESRI©), http://www.esri.com/arcgis/) software package, version 10.3.1. The plots (Fig.S2d,h) were created using the GMT (Generic Mapping Tools, http://gmt.soest.hawaii.edu/) software package[7], version 4.5.14.

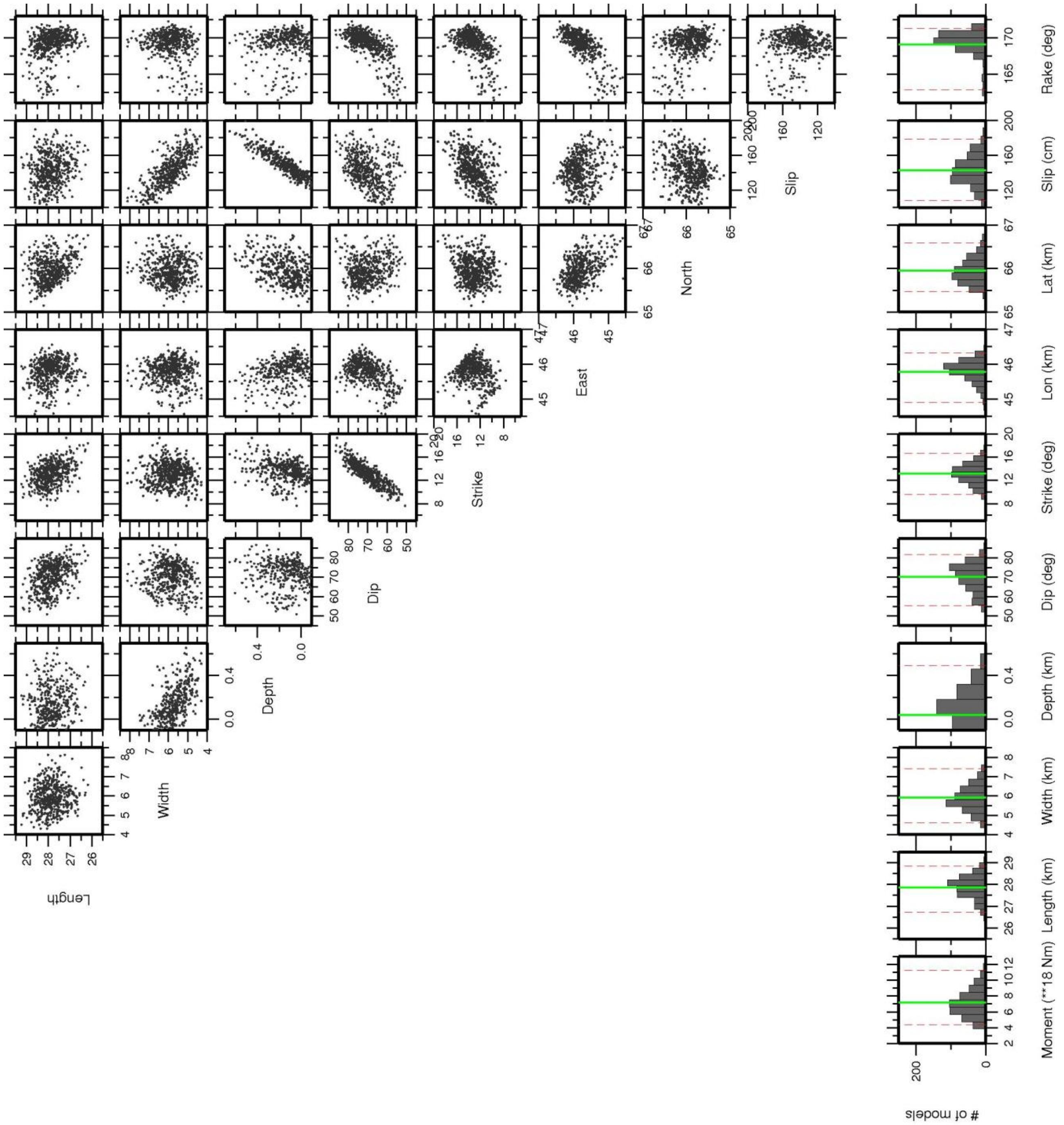

Supplementary Figure S3: Trade-offs between the model parameters of the uniform-slip dislocation model. The trade-offs are obtained by applying the best fitting technique to a large number (500) of synthetic data sets, each one derived from adding synthetic realizations of data noise to the actual data set. The bottom row (histograms) shows the *a posteriori* distribution of the model parameters (the red dashed lines bracket the 95% individual confidence intervals, while the green lines show the optimal parameter values). The other rows (scatter plots) represent the correlations between parameter pairs. The plot was created using the GMT (Generic Mapping Tools, http://gmt.soest.hawaii.edu/) software package[7], version 4.5.14.

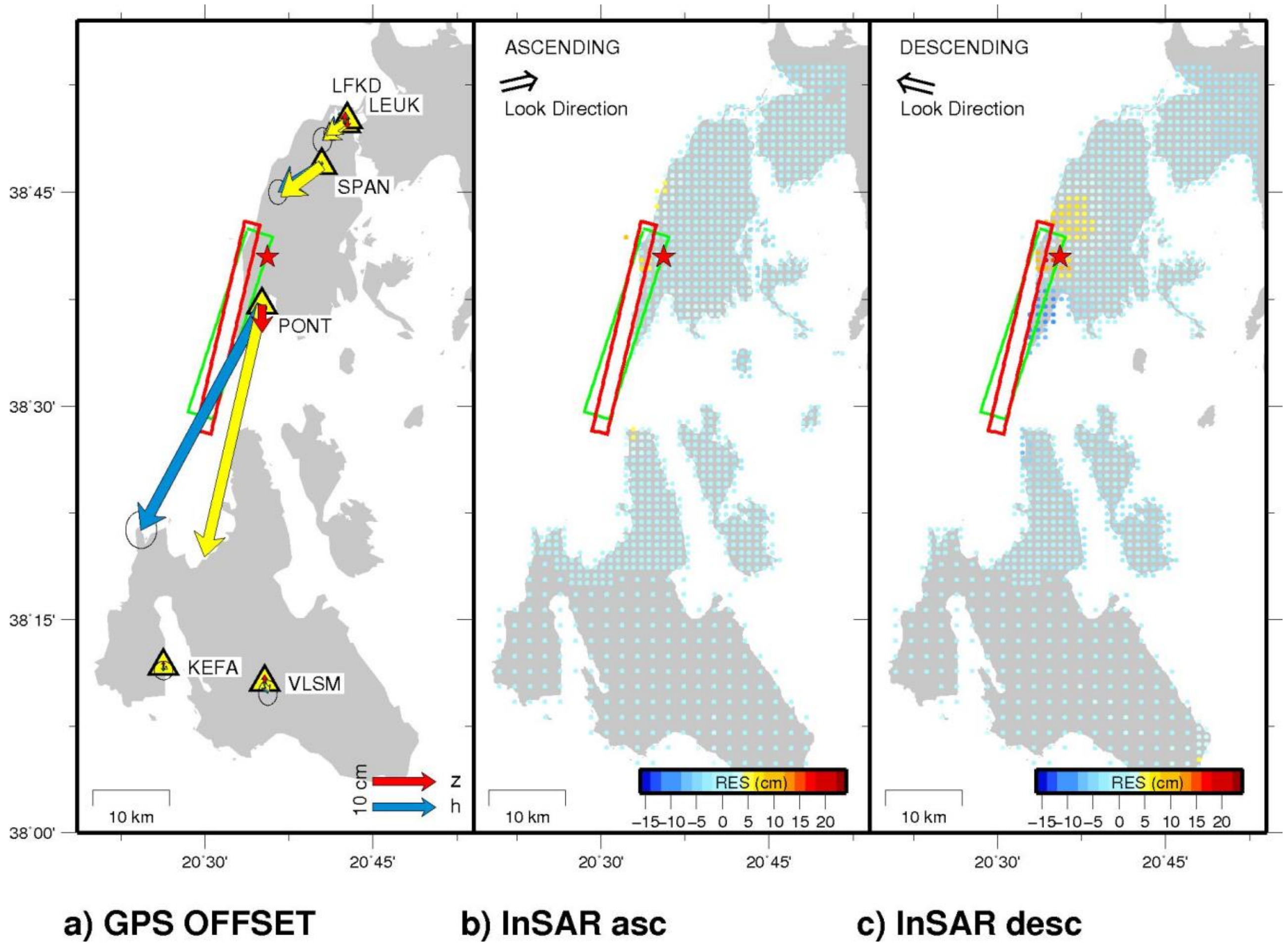

Supplementary Figure S4: Residuals of the uniform slip finite fault modelling. (a) Modeling of the finite fault with uniform slip projected to the surface (the red box) and comparison of measured (blue and red arrows) with synthetic (yellow and green arrows) horizontal and vertical coseismic static displacements. (b, c) Residuals between the observed and synthetic SAR displacements, concerning both ascending (center) and descending (right) Sentinel1-A interferograms. The green box is the seismic fault as determined by Ganas *et al.*[1]. The other symbols are the same as in Fig. 2 of the main text. Map was created using the GMT (Generic Mapping Tools, http://gmt.soest.hawaii.edu/) software package[7], version 4.5.14.

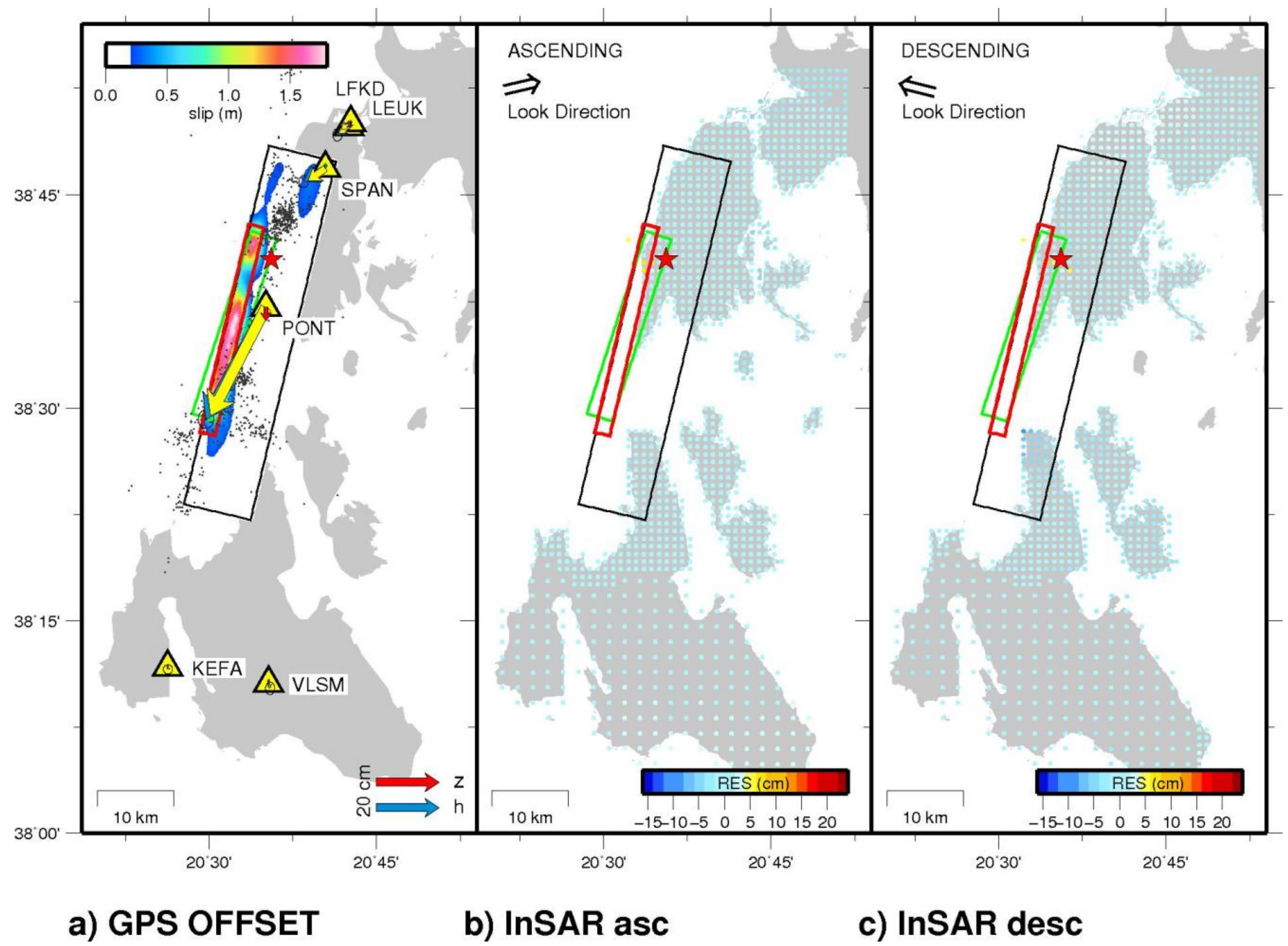

Supplementary Figure S5: Residuals of the variable slip finite fault modelling. (a) Modeling of the finite fault with variable slip projected to the surface and comparison of measured (blue and red arrows) with synthetic (yellow and green arrows) horizontal and vertical coseismic static displacements. Black dots represent the 956 relocated aftershocks in the time period 2015/11/17-2015/12/30[1]. (b, c) Residuals between the observed and synthetic InSAR displacements, concerning both ascending (center) and descending (right) Sentinel1-A interferograms. The red and black boxes are our uniform-slip (Fig. S4) and variable slip models respectively, whereas the green one is the seismic fault determined by Ganas *et al.*[1]. The other symbols are the same as in Fig. 2 of the main text. Map was created using the GMT (Generic Mapping Tools, http://gmt.soest.hawaii.edu/) software package[7], version 4.5.14.

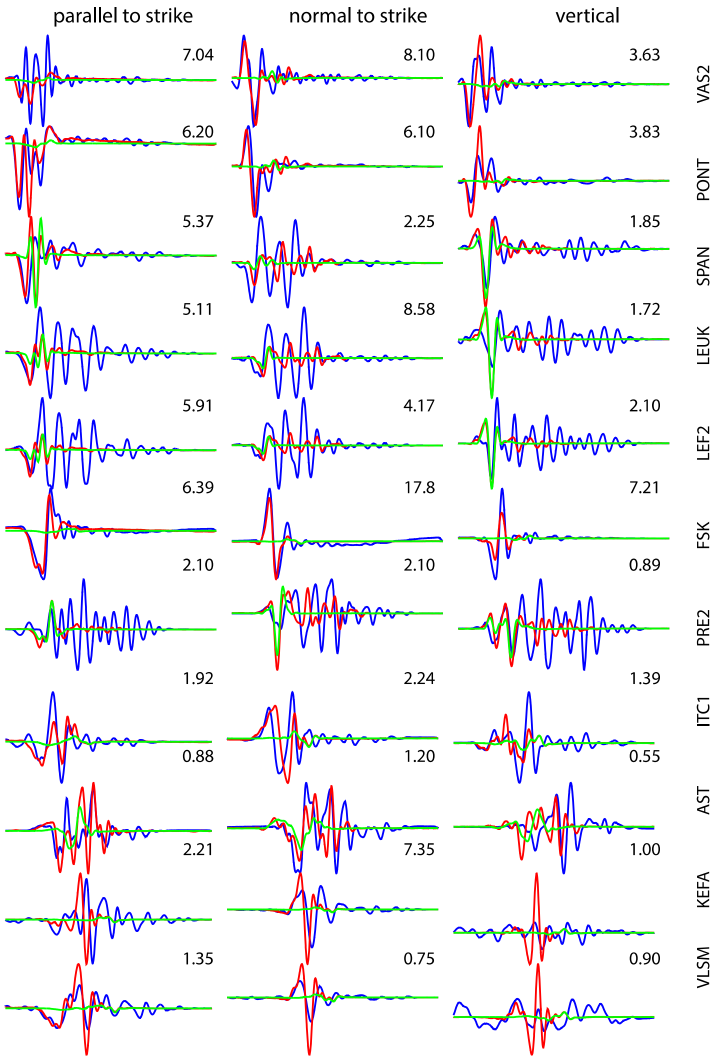

Supplementary Figure S6: Forward modeling of the NE slip patch. The figure shows the comparison between the observed, modeled, and predicted waveforms (blue, red, and green lines, respectively), the latter as predicted by taking into account the small third patch, located to the NE part of the fault plane, characterizing the rupture model retrieved from the joint inversion (Fig. 3a in the main text). Peak amplitudes (cm*s^{-1}) of the observed waveforms are given by numbers. The plot was created using the GMT (Generic Mapping Tools, http://gmt.soest.hawaii.edu/) software package[7], version 4.5.14.

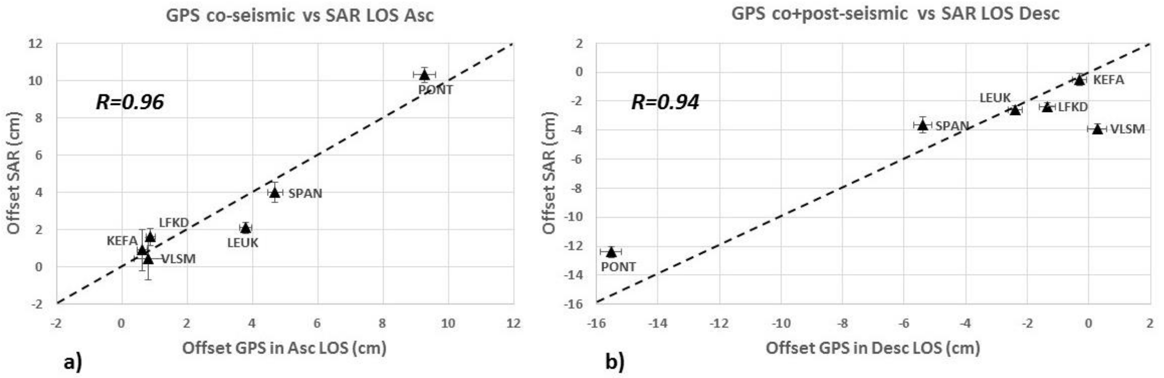

Supplementary Figure S7: Comparison between GPS-derived offsets and SAR LOS displacements. Comparison between "co-seismic" displacements and the ascending average LOS values (a) and between the "co+post-seismic" offsets and descending average LOS values (b). The R value correspond to the correlation coefficient. Horizontal and vertical error bars correspond to the uncertainties of GPS and SAR displacements, respectively. The plots were created using the Microsoft Excel 2010 package.

References

1. Ganas, A. *et al.* Coseismic deformation, field observations and seismic fault of the 17 November 2015 M=6.5, Lefkada Island, Greece earthquake. *Tectonophysics*, **686**, doi:10.1016/j.tecto.2016.08.012 (2016).
2. Sarmap (2012), SARscape technical decription, http://www.sarmap.ch/pdf/SARscapeTechnical.pdf
3. Okada, Y.,Surface deformation due to shear and tensile faults in a half-space. *Bull. Seism. Soc. Am.*, **75**, 1135-1154 (1985).
4. Corana, A., M. Marchesi, C. Martini, and S. Ridella Minimizing multimodal functions of continuous variables with the "Simulated Annealing" algorithm. *ACM Trans. Math. Softw.,* **13**, 262-280 (1987).
5. Press, W.H., S.A. Teukolsky, W.T. Vetterling, and B.P. Flannery Numerical Recipes in C: The Art of Scientific Computing, *2nd edn, Cambridge University Press, Cambridge* (1992).
6. Stark, P. B. & Parker, R. L. Bounded-variable least-squares algorithm – An algorithm and implications. *Comput. Stat.* **10**, 129-141 (1995).
7. Wessel, P. & Smith, W. H. F. New improved version of the generic mapping tools released. Eos. Trans. AGU 79, 577-579 (1998).
